# Pheochromocytomas and Paragangliomas: Clinical and Genetic Approaches

**DOI:** 10.3389/fendo.2015.00126

**Published:** 2015-08-17

**Authors:** Marcia Helena Soares Costa, Tania M. Ortiga-Carvalho, Alice Dutra Violante, Mario Vaisman

**Affiliations:** ^1^Division of Endocrinology, Federal University of the State of Rio de Janeiro, Rio de Janeiro, Brazil; ^2^Laboratory of Translational Endocrinology, Carlos Chagas Filho Biophysics Institute, Federal University of Rio de Janeiro, Rio de Janeiro, Brazil; ^3^Division of Endocrinology, Federal University of Rio de Janeiro, Rio de Janeiro, Brazil

**Keywords:** pheochromocytomas, MEN2, succinate dehydrogenase, VHL, neurofibromatosis

## Abstract

Pheochromocytomas (PCCs) and paragangliomas (PGLs) are neuroendocrine tumors derived from the chromaffin tissue. Diagnosis of these tumors is extremely important as they are linked to the hypertension syndrome with great cardiovascular morbidity and mortality. A great majority of PCCs and PGLs are sporadic and benign tumors; however, the classic idea of 10% exception of these features is changing. The description of new genes linked to familial forms of PCC/PGLs, such as succinate dehydrogenase (SDH) complex subunits, *KIF1B*β, *EGLN1*, *TMEM127*, and *MAX*, added to the well-known PCC familial syndrome (MEN2, VHL, and neurofibromatosis type 1) presents new challenges for diagnosis. In this review, we discuss the diversity of clinical and genetic approaches to this syndrome as well the diverse criteria that should guide genetic investigation.

## Introduction

Pheochromocytomas (PCCs) and paragangliomas (PGLs) are neuroendocrine tumors derived from the chromaffin tissue of the adrenal medulla or from extra-adrenal sympathetic and parasympathetic paraganglia. Parasympathetic PGLs are mostly non-secreting, whereas sympathetic PGLs generally produce catecholamine (1, 2).

The diagnosis of these neoplasias is extremely important, as they are associated with hypertension syndrome and great cardiovascular morbidity and mortality. The prevalence of PCC/PGLs in general hypertensive population is on average 0.1–0.6% and even higher in children (1.7%), with 5% of incidentally discovered adrenal mass (1, 3, 4). However, with modern imaging, 20–30% of these tumors are incidentally discovered (5). Therefore, high suspicion is necessary to avoid misdiagnosis.

## Clinical Diagnosis Based on Genetic Aspects

The American Endocrine Society guidelines advocate that it is important to be aware of the main clinical features for proper diagnosis of PCC/PGLs (5). The clinical presentation of these tumors can overlap with other clinical conditions with non-specific symptoms, such as tachycardia, diaphoresis, pallor, anxiety, headaches, panic attacks, and sensation of sudden death. Hypertension may or may not be present; as previously described by an Italian group where 21% of patients with PCC were normotensive, and it can appear as a sustained sign or during paroxysm (6). In humans, blood pressure depends on many factors including the sympathetic nervous system and the renin–angiotensin–aldosterone system among other factors. In patients with PCC, however, the behavior of hypertension depends also on the circulating catecholamine levels and eventually by other cardiovascular active substances secreted by the tumor. Moreover, production of vasodilator substances, such as dopamine or prostaglandins, downregulation of alpha-1 adrenergic receptors, and the presence of hypovolemia can contribute to this variability. Norepinephrine (NE)-secreting tumors usually cause sustained hypertension, while tumors that produce epinephrine (E) co-secreted with NE commonly result in episodic hypertension. Pure E-secreting tumors can present hypotension instead of high pressure as a symptom (7). Large cystic tumors are often asymptomatic because part of the catecholamine produced is metabolized inside the tumors before being released into circulation (8).

**Figure 1:**
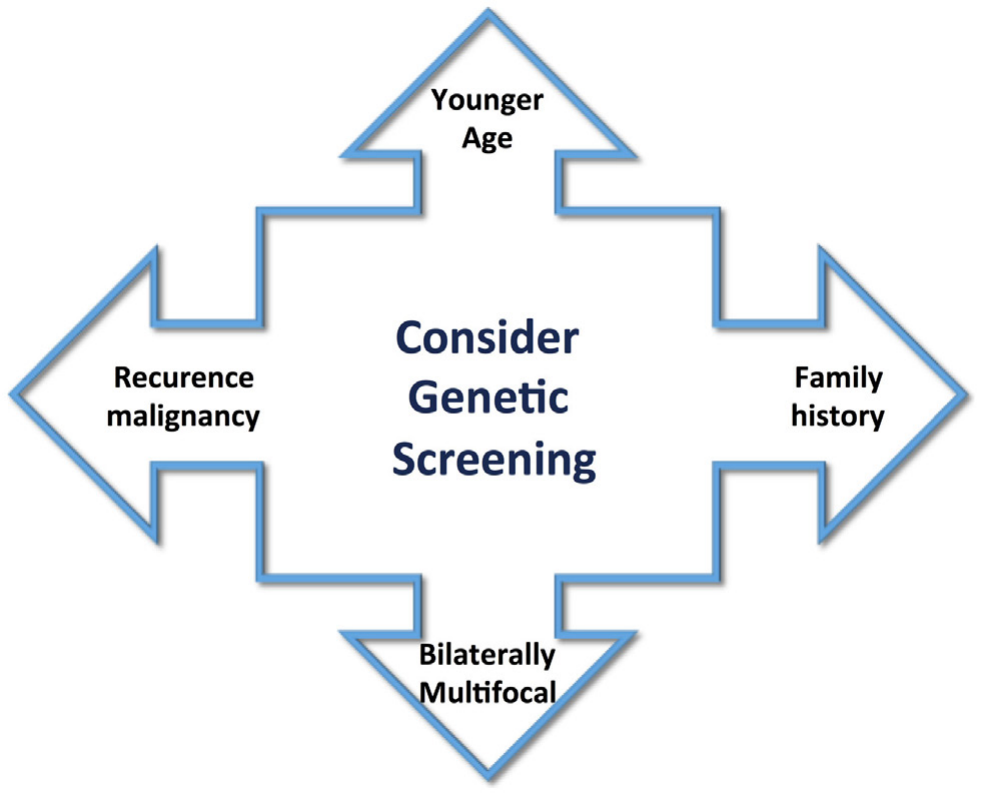
**Clinical aspects to guide the screening for genetic abnormality include younger age of tumor appearance, positive family history, bilaterally multifocal tumors, and recurrence or malignancy**.

The heterogeneity of PCCs/PGLs clinical presentation explains the reason why this disease frequently is not considered as a possible diagnose condition, though can provoke a life-threatening crisis. At that point, any patient with the clinical manifestations cited before should be included as a case suspicion and must be properly screened for PCCs. These tumors occur in any age, but predominantly between 40 and 50 years, with approximately equal sex distribution (9, 10). However, the age of appearance can change in hereditary tumors, and they often occur at an earlier age. A great majority of PCCs and PGLs are sporadic and benign tumors; however, the classic idea of the traditional 10% rule for these features is changing. Occurrence of hereditary-associated tumors has increased to 30%. Also, diagnosis of several syndromes, such as multiple endocrine neoplasia type 2 (MEN2), von Hippel–Lindau disease (VHL), and neurofibromatosis type 1, has increased. Recently, additional mutations in the succinate dehydrogenase (SDH) complex subunits, *SDHA*, *SDHB*, *SDHC*, *SDHD*, and *SDHFA2*, related to familial PCC–PGL and novel susceptibility genes, such as *KIF1B*β, *EGLN1*, *TMEM127*, and *MAX*, have led to the diagnosis of new phenotypes (11, 12). Patient criteria, such as younger age, positive familial history for PCC/PGL, tumor bilaterally/multifocal, and recurrence or malignancy, could guide selection of patients who may be under greater risk of genetic abnormality (Figure 1). Genetic tests are expensive, and therefore, the knowledge of links between specific clinical and biochemical phenotype and PCC/PGLs-related genes and syndromes could reduce the cost of genetic screening significantly. This review aims to provide an overview of the genetic susceptibility of these tumors, clarifying and reinforcing the importance of their diagnosis.

## Genes and Syndromes Related to PCC/PGL

### Multiple endocrine neoplasia

Multiple endocrine neoplasia type 2 is described by medullary thyroid carcinomas (MTC) in association with PCC. This syndrome has three clinical variants: MEN2A (55% of cases), familial medullary thyroid carcinoma (FMTC – 35–40%), and MEN2B (5–10%) (13). The disease is caused by the germ-line mutation of the proto-oncogene, *RET* (rearranged during transfection). This 21-exon proto-oncogene is located on chromosome 10 and encodes a receptor tyrosine kinase that works in conjunction with glial-derived neurotrophic factor family ligands. The interaction of these ligands results in the dimerization of the *RET* receptor, auto-phosphorylation of tyrosine residues, and finally cell growth and survival mediated by the mitogen-activated protein kinase intracellular signaling cascade. One germ-line activating mutation of this gene, generally missense and located in exons 10, 11, 13, 14, 15, 16, has been implicated in this syndrome (14, 15). MEN2A is also associated with hyperparathyroidism in 20–30% of the patients, unlike MEN2B, in which this feature is absent and marfanoid habitus calls attention. MEN2B tumors are more aggressive, with an earlier onset and higher mortality.

MEN2 types carry approximately 100% risk for MTC and at 50% risk for PCC. FMTC tumors are less aggressive and, by definition, without incidence of other endocrine neoplasia. There is a strong correlation between the phenotype and genotype in the clinical subtypes of MEN2 with respect to age of onset and aggressiveness of MTC. These aspects are used to determine the time of prophylactic thyroidectomy and whether patients should be screened for PCC and hyperparathyroidism. In this way, positive results for a specific *RET* mutation can influence the management of an apparently sporadic MTC.

Pheochromocytomas in MEN2A and B are usually benign, bilateral in half of the patients, and can rarely be extra-adrenal. The malignancy affects <5% of PCCs in this syndrome (16, 17). Mutation in codons 618, 620, and 634 of *RET* is representative of MEN2A in approximately 80% of patients. A single missense mutation in codon 918 is found in nearly 90% of MEN2B cases. Based on this genotype and phenotype correlation, the American Thyroid association has proposed the classification of four risk-categorized mutations and recommends the screening for PCC by the age of 20 years for MEN2A and by 8 years for MEN2B (18). One group has reported a high penetrance of PCCs, especially bilateral and large tumors, associated with a double C634/Y719F germ-line mutation in the *RET* protooncogene (19).

### Von Hippel–Lindau syndrome

Von Hippel–Lindau syndrome is an autosomal dominant disease associated with inactivating mutations in the *VHL* tumor suppressor gene located on chromosome 3. This disease is characterized by a multiplicity of benign and malignant tumors, including retinal and central nervous system (CNS) hemangioblastomas, pancreatic islet cell tumors and pancreatic cysts, epididymal cystadenomas, endolymphatic sac tumors, clear-cell renal carcinomas and PCCs, and PGLs (20–23).

This syndrome is divided into type 1 or type 2 based on the absence or presence of PCC, respectively. The VHL type 2 is further classified as 2A for patient with low risk of developing clear renal cell carcinoma, type 2B for patients with high risk of these tumors, and 2C for patients with only PCCs in the absence of the others classical tumors of the syndrome (23, 24). *VHL* mutations result in unilateral or bilateral PCCs and rarely in PGLs. Approximately half of the cases are bilateral and benign tumors, with malignant phenotype present in <5% of the patients (10, 25). The mean age at diagnosis of these tumors is in the second decade, generally approximately 30 years, and the absence of signs and symptoms is frequent. PCCs and PGLs are the first manifestation in approximately 30–50% of the cases. Due to these aspects, it has been proposed that catecholamine screening should be started at approximately 5 years old, especially in patients with a positive familial history of PCCs (12, 26).

There are some correlations between the genotype and the phenotype in this syndrome. The *VHL* gene comprise three exons, and its mutations are distributed throughout the coding sequence; missense, nonsense, splice-site mutations, micro deletions, and insertions are found in two-thirds of patients, while large deletions are described in only one-third of patients with this syndrome (20, 27). Deletions, nonsense, and frame shift mutations are regularly found in VHL type 1, whereas missense mutations are more common in VHL type 2. A majority of patients with PCC have missense mutations. Maher et al. (20) analyzed the genotype–phenotype correlations in 573 individuals with VHL disease and demonstrated that classifying missense substitutions according to their predicted effect on pVHL structure enhances the ability to predict pheochromocytoma risk. Although genetic screening has been able to identify mutations in the majority of patients with VHL disease, some patients (20%) without genetic diagnosis probably develop *de novo* mutations (28).

### Neurofibromatosis type 1

Neurofibromatosis type 1 (NF1) or Von Recklinghausens’s disease is an autosomal dominant syndrome characterized by *café au lait* macules, cutaneous or subcutaneous neurofibromas, skinfold freckling, and iris Linch nodules. These characteristics occur in an average of 90% of all NF1 adult patients, but the number of lesions is quite variable. Several other tumors, such as nerve glioma, dysplasia of the long bones, peripheral nerve sheath tumors, gastrointestinal stromal tumors (GIBT), rhabdomyosarcomas, breast cancer, and chronic myeloid leukemia, have also been described. Some patients can also present cognitive impairment (29, 30).

Pheochromocytomas are a rare finding in NF1, being identified in <6% of cases; however, they have a higher prevalence (13%) in autopsy series. The preferential mean age of presentation is in the fourth decade. A majority of these PCCs are unilateral and benign, while approximately 10% can be bilateral or malignant. The presence of PGLs is not common in NF1 (31, 32). Considering these aspects, the main features of PCCs in NF1 are very similar to those of sporadic PCCs.

*NF1* is a tumor suppressor large gene with 60 exons located on chromosome 17 with described missense, nonsense, and splice-site mutations, indels, and chromosomal rearrangements (33). These mutations are spread all over the gene without a hot spot, and the majority are located in the exons with variable penetrance and expression (32).

The *NF1* protein, neurofibromin, is a GTPase that suppresses cell proliferation by inactivating RAS and inhibiting the RAS/RAF/MAPK signaling pathways. When this gene is mutated, the cascade including PI3K and mTor is activated, subsequently resulting in uncontrolled cellular growth and differentiation (34). Some studies have suggested a possible novel potential NF1 genotype–phenotype correlation, especially with splice-site mutations associated with an increased tendency to develop neoplasms as CNS gliomas and malignant peripheral nerve sheath tumors (12). However, the diagnosis of the NF1 is established mainly based on the clinical aspects because it is difficult to genetically screen in the absence of hot spot mutations.

### Familial PCC/PGLs

#### SDHx-Associated PCCs and PGLs

In the last decade, the genetics of hereditary PCC/PGLs has started to emerge with the description of germ-line mutations in the SDHx (SDH) genes related to this syndrome. The SDH complex is described as a heterotetrameric protein involved in the electron transport in the Kreb’s cycle and mitochondrial respiratory chain. This enzyme is composed of four subunits (SDHA, SDHB, SDHC, and SDHD) that catalyze the oxidation of succinate to fumarate. The catalytic subunits, SDHA and SHDB, are inserted in mitochondrial matrix, and they are affixed to the inner membrane by subunits SDHC and SDHD. The SDHx genes are supposed to work as classical tumors suppressors, as tumors frequently show loss of heterozygosity (LOH) of the non-mutated allele.

The relations between SDH and PCC/PGLs emerged in the 2000s when germ-line *SDHD* mutations were associated to the occurrence of PCC/PGLs (35). *SDHD* is located on chromosome locus 11q23, has four exons, and is maternally imprinted (36, 37). The disease susceptibility occurs when the mutation is inherited from the father with rare exceptions.

*SDHD* mutations are linked to the development of familial PGLs and PCCs (PGL1), although they have been reported in apparently sporadic tumors; they are often associated with the risk of multiple tumors, especially head and neck PGLs (38, 39). PCC can occur usually unilateral (40). Malignancy associated with SDHD-derived PCC/PGL is quite rare.

The PGL2 syndrome is associated with *SDHAF2* mutations, also known as SDH5. The *SDHA* mutation has been described with Leigh’s disease, a severe type of encephalopathy. The *SDHA* maps to chromosome 5p15, its products undergo post-translational modification by SDHAF2, and the gene of this protein has been implicated in development of hereditary head and neck PGLs (41, 42). Mutations in *SDHAF2* are considered rare; in a study with 443 patients with head and neck PGL evaluated, mutations on the *SDHAF2* gene were negative.

Mutations in *SDHC* are implicated in PGL3 syndrome. The gene is located on chromosome 1q23.3 and presents six exons. A mutation in *SDHC* is less frequent when compared to *SDHD* mutations; it is usually associated with benign solitary head and neck PGLs. Also, extra-adrenal PGLs and PCCs have been described in this syndrome. Malignancy associated with the *SDHC* gene is extremely rare (43).

Mutation in *SDHB* is implicated in PGL 4 syndrome, characterized mostly by abdominal, pelvic, and thoracic catecholamine-secreting familial PGL. The *SDHB* gene is located on chromosome 1p36.1, with eight exons. The risk of malignancy is great with this disease; less frequently, these mutations are found in PCCs or parasympathetic PGLs. The associated risk of malignancy is difficult to determine, especially because metastases can occur up to 20–30 years after primary tumors, and the applied definitions of malignancy vary between different studies (44).

Furthermore, *SDHB* mutations may result in susceptibility to other malignant tumors, such as renal cell carcinomas, papillary thyroid tumors, neuroblastoma, or gastrointestinal stromal tumor (GIST). Average 20% of patients with *SDHB* mutation will present malignant PGLs, and more than 50% of the individuals with malignant PGLs will have a *SDHB* mutation (40, 44). Recently, mutations in this gene and in *SDHD* and *SDHC* were described in association with Carney–Stratakis syndrome, an autosomal dominant disorder characterized by PGLs and GIST, but they have not been found in other forms of the Carney triad, suggesting that other genetic alterations may be implicated in this syndrome (45).

More efforts are needed to define the risk of other cancers in association with these mutations in order to help clinicians better design a phenotype and genotype correlation in PCC\PGLs and understand the complexity of these diseases.

#### Carney Triad and Carney–Stratakis Syndrome

Carney triad is a syndrome of tumors involving at least five organs, the stomach, the lung, the paraganglionic system, the adrenal (cortex and medulla), and the esophagus. The stomach features GIST, the lung chondroma, the paraganglionic system paraganglioma, the adrenal adenoma and pheochromocytoma, and the esophagus leiomyoma (46).

The etiology of the Carney triad syndrome is unknown; no candidate gene has been discovered until now and the disease does not appear in a family pattern. The younger age and female predilection of affected individuals do implicate in a link with an inherited genetic defect; however, the screening of *SDHA, SDHB, SDHC*, and *SDHD* mutations has been always negative. The mutated genes, often associated to GIST (*KIT* and *PDGFRA*), are also not affected.

Differently, Carney–Stratakis syndrome is inherited in an autosomal-dominant manner that includes PGLs and GIST without pulmonary chondromas (47). This disease affects equally men and women and the majority of patients present germ-line mutations in *SDHB, SDHC*, or *SDHD* supporting another molecular mechanism linked to GIST besides KIT and PDGFRA mutations usually described (48).

The associated PCC and PGLs in these syndromes can be different. In Carney triad disease, the majority of patients present with PGLs, including sympathetic and parasympathetic tumors, with 28 years old of mean age presentation; 11% of the patients present metastasis (49). In Carney–Stratakis syndrome, the majority of patients have a sympathetic or parasympathetic PGLs, with a mean age of 33 years old; however, no malignant tumors were described (47).

#### New PCCs and PGLs Susceptibility Genes

Despite identification of multiple genetic causes of PGLs and PCCs in the last decade, around two-thirds of these tumors remain without molecular diagnosis, suggesting that other susceptibility genes could be implicated. Using a wide-genomic approach, PCC/PGLs were clustered in two major groups depending on their global transcription profiles. Cluster 1 includes *VHL*, SDHx, FH-mutated tumors and a part of the sporadic PCC/PGLs. This group was composed of tumors without clear mutation or sporadic tumors; they showed signatures of pseudo hypoxia, angiogenesis, and decreased oxidoreductase response. This profile links these tumors with hypoxia-inducible factor (HIF) role, and overexpression of HIF1α and 2α were found in this cluster.

A second group (cluster 2) covered PCCs with *RET*, *NF1*, *KIF1B*β, and undefined tumors enriched for kinase receptor signaling pathways, translation initiation, protein synthesis, and genes involved in neural/neuroendocrine identity (50–52). Novel genes, such as *TMEM127*, were also identified in this cluster (53). Also, deregulation of another pathway involving egl nine homologs was suggested (54).

*KIF1*β is a tumor suppressor gene necessary for neuronal apoptosis. It is located on chromosome 1 (55). Mutation in this gene was described in patients with PCC without other predisposing mutations. No specific phenotype has been identified yet; however, patients with *KIF1*β seem to be prone to PCC and neuroblastomas. Ganglioneuromas, leiomyosarcomas, and lung adenocarcinomas have also been reported (50).

In 2008, a germ-line mutation in *EGLN1*, also called *PHD2*, was reported in a patient with erythrocytosis and recurrent para-aortic PGL. *EGLN1* is a member of EGLN prolyl hydroxylase family that plays a major role regulating the HIF, which is involved in angiogenesis, erythropoiesis, cell metabolism, and proliferation. This gene was linked to familial erythrocytosis but not in association with tumors. A mutation analysis of 82 patients with inherited PCC did not detect mutations in *EGLN1*, *EGLN2*, or *EGLN3*, suggesting that mutations in these genes are not a frequent cause of inherited PCC (56).

*TMEM127* was recently identified as a PCC/PGLs susceptibility gene using linkage analysis and transcription profiling by microarray and copy number analysis of these tumors. It is possible that *TMEM127*, located on chromosome locus 2q.11.2, could be a tumor suppressor gene playing a role in cell signaling associated with kinase receptor signals and as a negative regulator of mTOR, which promotes cell growth and protein translation (53). The most common clinical presentation of the mutation carriers is quite similar to sporadic PCC patient, single adrenal tumor with mean age of 40 years (57, 58), although multiple head and neck PGLs and retroperitoneal PGLs have been described. Unilateral and bilateral tumors have also been found, but malignancy is rare.

MYC-associated factor X (MAX) is a transcription factor that was recently linked to PCC/PGL susceptibility. *MAX* is located on chromosome locus 14q23 and was associated with other cancers, such as neuroblastomas, acting as an oncogene or interacting with MYC family as a transcriptional suppressor (59, 60). Whole genome sequencing identified MAX germ-line mutations in familial cases of PCC without alterations in known genes. Comino-Mendez et al. studied 12 PCC patients with *MA*X mutations; three of the cases were unrelated individuals with hereditary PCC (59). The absence of MAX protein in the tumors and LOH of the wild-type allele in these tumors confirmed the tumor suppressor role of this gene in humans. Eight of these patients (67%) had bilateral PCCs, and 25% presented metastasis at diagnosis, suggesting that *MAX* mutation can be associated to multicentricity and malignant risk.

## Genes and Sporadic PCC/PGLS

A problem that concerns clinicians is whether all sporadic PCCs should be screened for unsuspected germ-line mutations in genes associated with hereditary PGLs or PCCs. This aspect is not well clarified in the literature; the studies usually not include the analyses of all genes implicated in the familial forms. In a review by Jiménez et al., the authors provide a compilation of several studies, and the results suggest that 20% of patients with apparently sporadic PCCs have a germ-line mutation of one of the genes known to cause hereditary PCC/PGLs. When the authors excluded patients with bilateral or muticentric tumors with larger probability of familial disease, the amount was reduced to 17% (5.04% for *VHL*, 6.38% for *PGL4*, 3.72% for *PGL1* e 1.55% for *RET*) (61).

In one study developed by Neumann et al., it was found that 24% of the patients with apparently non-syndromic pheochromocytomas and no family history of the disease had mutations in *VHL*, *RET*, *SDHD*, and *SDHB* genes; these patients presented at a younger age (average of 24 years), and they had multiple tumors and 28% extra-adrenal tumors (62). Another study developed by the same group reinforced that up to 35% of patients with PCC/PGLs can be associated to an inherited mutation in these genes (32).

A Spanish group analyzed 135 apparently sporadic patients with a single tumor for the five susceptibility genes: *VHL*, *RET*, *SDHB*, *SDHC*, and *SDHD*. They found that 14% of the cases harbor germ-line mutations; a majority (98%) of them had tumors with younger onset, not more than 45 years old (63).

Demographic, clinical, and genetic evaluation was performed in a series of 71 patients with PCC/PGLs. Among 59 patients with apparently sporadic disease, unsuspected germ-line mutations occurred in 8 cases (13.6%). The authors did not find any difference between hereditary and sporadic disease concerning age, sex, and tumor size; bilateral and recurrent diseases were most common in hereditary cases (64).

Currently, the overall hereditary predisposition of PCCs is estimated to be approximately 20–35%. The high prevalence of unsuspected mutations indicates a demand for more extensive genetic tests for patients with these tumors (65). However, we have to be aware of the aspects that can influence the prevalence of the PCC/PGL mutations: tertiary care centers are more prone to evaluate patients with multiple and more complicated PCCs. Specific genetic disorders are more prevalent in some geographic regions, and major effort is being currently contributed to easier recognition of these syndromes. An Endocrine Society taskforce that compiled 31 studies including 5031 patients with apparently sporadic PCC/PGLs screened for numerous genes, and the taskforce reinforces this idea, as they found approximately 11–13% germ-line mutations in this population (66).

Somatic mutations in sporadic pheochromocytoma/paraganglioma has been identified in *VHL*, *RET*, *SDHB*, and *SDHD* but their frequency was reported to be low. The COMETE cohort has demonstrated the occurrence of somatic mutations in VHL and RET genes in 14% of sporadic pheochromocytoma/paraganglioma, one germ-line, and three somatic mutations in the *MAX* were described by the same cohort (67, 68). Burnichon et al. have found that 41% (25/61) of the sporadic tumors in their population harbor an inactivating somatic mutation in the NF1 gene associated with loss of the wild-type allele in 84% (21/25) of cases (69). These findings reinforce that somatic mutation in sporadic PCC/PGLs can be more often than previously considered.

## Genetic Screening Strategy in Patients with PCC and PGL

The American Society of Clinical Oncology suggests that all patients with PCC and PGLs should be submitted to genetic screening. It is currently accepted from the literature that approximately 30–35% of the PCC/PGLs are associated with germ-line mutations in one of the genes described above (70).

Molecular analysis has revealed that a significant number of PCC/PGLs, previously considered as sporadic tumors, have a genetic predisposition. However, in the last decade, several new genes were implicated in this disease, and we have considered the cost and availability of genetic tests before using them as diagnostic tools in PCC/PGLs.

Some characteristics, including personal or family history of PCC/PGLs, are very important. A detailed and medical family history and sometimes a specialized genetic consultation provide the potential consequences of genetic testing. In the absence of any family history, the description of sudden death without other cardiovascular disease should call attention. Clinical manifestations in the patient or other family members can point to a particular hereditary condition. For example, subtle retinal vascular lesions can indicate the possibility of VHL disease.

Generally, hereditary tumors occur at a younger age than the sporadic ones; age of diagnostics younger than 40 years is an ­important factor to call for a genetic test. An interesting study suggests that patients with epinephrine-producing tumors (mean age 50 years) were diagnosed 11 years later than those with tumors lacking appreciable epinephrine production (mean age 42 years). This finding reinforces that the cut-off age for genetic screening could consider tumor catecholamine phenotypes (71). However, finding nearly 36% germ-line mutations in children make these tests necessary in this group of patients (72). Presence of head and neck PGLs, bilateral or multifocal tumors, and the presence of malignancy should raise the suspicion of a possible hereditary abnormality (73, 74).

**Figure 2:**
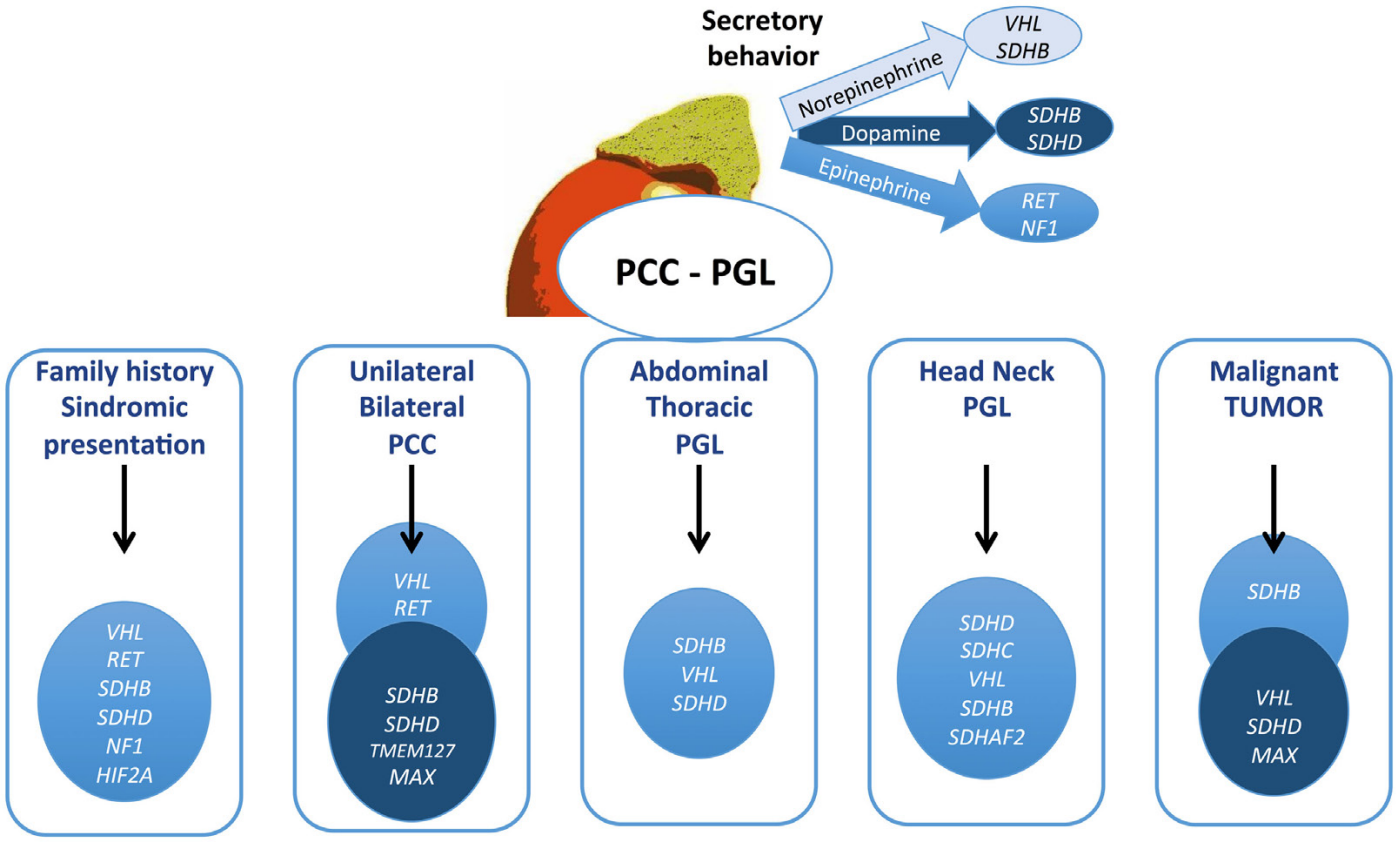
**Algorithm proposed for genetic testing for pheochromocytomas and paragangliomas patients**. The gene described might be considered for testing in this order. Mutations in *TMEM127*, *MAX*, *HIF2A*, and *SDHAF2* are quite rare and should be contemplated in patients with negative test for the other genes.

For family diseases, such as MEN2A/B, NF1, and VHL, a phenotype suspicion, because they are commonly associated to other tumors, helps to direct to genetic studies. The presence of hemangioblastomas calls attention for VHL disease, as the association of medullary thyroid carcinoma for MEN2. MEN2-associated tumors always produce epinephrine and can be uni- or bilateral, but malignant disease and extra-adrenal tumors are rare in this syndrome. PCCs in VHL always secrete NE and can be extra-adrenal. The biochemical profile, as shown in Figure 2, is very relevant to help and guide the genetic test. Diagnosis of NF1 is based on clinical features, and genetic testing is usually not required, because *NF1* is a large gene, costly, and difficult to screen.

Adrenal (unilateral or bilateral) PCCs are frequently associated with VHL and RET, while extra-adrenal sympathetic PGLs (abdominal or thoracic) are usually caused by *SDHB*, *SDHD*, and *VHL* mutations (75–77). Head and neck tumors are mainly caused by SDH mutations, especially *SDHD* and B, and less often by *VHL* and *SDHC* (25, 40). *SDHC* mutations have been described exclusively in patients with parasympathetic PGLs and quite rare in association with sympathetic PCC/PGLs (43). Tests for this gene are only indicated in these cases. Genetic test for *SDHB* is indicated in all patients with malignant PGL or PCC, and up of 50% of these tumors have an SDHB mutation. Elevation of dopamine with NE has been found in these tumors. Unlike classical syndromes, only 10% of the SDHB patients have a positive family history.

*TMEM127* is customarily associated with benign PCC but not with PGL. Ordinarily, these tumors occur in old age, mimicking a truly sporadic PCC (57, 64).

Routine screening for germ-line mutations in patients with PCC/PGLs is costly and time consuming. Although some algorithms have been proposed, no routine guideline is available (73). For these reasons, the genetic screening directions are mostly based on clinical expertise, including clinical signs, the biochemical phenotype, and symptoms (Figure 2; Table 1). Understanding the genetic profile of these tumors and considering the patient’s risk for developing multifocal lesions, malignant PCC/PGLs, and other malignancies provide a very important tool for following patients with this condition. Regarding these aspects, we suggest to screening all patients with multiple tumors, especially with extra-adrenal and head and neck tumors. Age is an important factor; nevertheless, some studies have demonstrated that the age range can be quite wide, even for familial forms. The genes might be screened according to clinical presentation; then cost and effective analyses, screening of *SDHB* and *VHL* should be considered. The most important clinical aspects related to different genes are provided in Table 1. Head and neck PGLs should be first screened for *SDHD* and *SDHC* mutations.

**Table 1:** **Clinical and genetic features of the most frequent pheochromocytomas and paragangliomas-related syndromes**.

Gene	Loci	Protein function	Syndrome	Clinical manifestations or associated TU	Inheritance	Primary location	Malignacy rate
*RET*	10q11.2	Transmembrane tyrosine kinase	MEN2A	MTC, Hyperparathyroidism, cutaneous lichen amyloidosis	AD	PCC (single ou bilateral)	<5%
			MEN2B	MTC, mucosal neuromas, marfanoid habitus			
*VHL*	3p25–26	Ubiquitin ligase 3E activity	VHL	Hemangioblastomas, clear-cell renal cell carcinomas; endolymphatic sac tumors, cystadenomas; pancreatic islet cell tumor	AD	PCC (>bilateral)	<5%
*NF1*	17q11.2	GTPase	NF1	Café au lait macules, neurofibromas, iris Linch nodules, optic nerve gliomas, dysplasia of the long bones, astrocytomas, soft tissue sarcomas, CML, childhood learning disabilities, seizures	AD	PCC (>single)	12%
*SDHD*	11q23.1	Complex II anchoring subunit	PGL1	Papillary thyroid cancer; GIST	AD; PI	HNPGL/MPGL	<5%
*SDHAF2*	11q13.1	Cofactor for complex II	PGL2	GIST	AD; PI	HNPGL/MPGL	Low
*SDHC*	1q23.3	Complex II-anchoring subunit	PGL3	GIST	AD	HNPGL > SPCC = TAPGL = MPGL	Low
*SDHB*	1p36.1	Complex II catalytic subunit	PGL4	Renal cell carcinomas; GIST, thyroid tumors, neuroblastomas	AD	TAPGL > HNPGL = SPGL = MPGL < SPCC	30–70%
*SDHA*	5p15	Complex II catalytic subunit			AD	TAPGL/HNPGL	Low
*TMEM127*	2q11.2	Transmembrane protein			AD	SPCC > BPCC > TAPGL = HNPGL = MPGL	Low
*MAX*	14q23–3	BHL HLZ transcription factor			ADPI	SPCC = BPCC > TAPGL	10%

*AD, autosomal dominant; PI, paternal inheritance; TAPGL, thoracic or abdominal PGL; HNPGL, head and neck PGL; SPCC/PGL, single PCC/PGL; BPC/PGL, bilateral PCC/PGL; MPGL, multiplal PGL*.

Currently, it is recommended that if a mutation is found, it is not necessary to search for additional genomic alterations; however, with the description of new genes, we have to be aware of new possibilities. Identification of a gene mutation in patients with PCC/PGLs results in precocious diagnosis and better surveillance, resulting in better prognosis. In specific cases, *RET* screening and genotype and phenotype correlation permits prophylactic surgery for an aggressive tumor. Nevertheless, further clinical studies are indispensable to better correlate genotype and phenotype features with the new genes.

## Conclusion

To guide better screening strategy and optimize therapeutic options, the knowledge of PCC/PGL-implicated syndromes and genes is incredibly important. This review summarizes the main syndromes and the gene associated with these diseases. Further studies are needed for wider genotype to phenotype correlations, especially for new genes and pathways recently described for these tumors.

## Author Contributions

MC, TO-C, AV, and MV contributed to the conception and design of the collection of the data for the revision, drafted the work, and critically revised the intellectual content. All the authors approved the last version of the manuscript and agreed to be accountable for all aspects of the work.

## Conflict of Interest Statement

The authors declare that the research was conducted in the absence of any commercial or financial relationships that could be construed as a potential conflict of interest.
